# In vitro antibacterial activity and cytocompatibility of magnesium-incorporated poly(lactide-*co*-glycolic acid) scaffolds

**DOI:** 10.1186/s12938-020-0755-x

**Published:** 2020-02-18

**Authors:** Rui Ma, Wei Wang, Pei Yang, Chunsheng Wang, Dagang Guo, Kunzheng Wang

**Affiliations:** 1 Department of Bone and Joint Surgery, the Second Affiliated Hospital of Xi’an Jiaotong University, Xi’an, 710004 Shanxi China; 2 State Key Laboratory for Mechanical Behavior of Materials, School of Materials Science and Engineering, Xi’an Jiaotong University, Xi’an, 710049 Shanxi China

**Keywords:** Magnesium, Antibacterial activity, Poly(lactide-*co*-glycolic acid), Infection, Scaffold

## Abstract

**Background:**

Bone defects are often combined with the risk of infection in the clinic, and artificial bone substitutes are often implanted to repair the defective bone. However, the implant materials are carriers for bacterial growth, and biofilm can form on the implant surface, which is difficult to eliminate using antibiotics and the host immune system. Magnesium (Mg) was previously reported to possess antibacterial potential.

**Methods:**

In this study, Mg was incorporated into poly(lactide-*co*-glycolic acid) (PLGA) to fabricate a PLGA/Mg scaffold using a low-temperature rapid-prototyping technique. All scaffolds were divided into three groups: PLGA (P), PLGA/10 wt% Mg with low Mg content (PM-L) and PLGA/20 wt% Mg with high Mg content (PM-H). The degradation test of the scaffolds was conducted by immersing them into the trihydroxymethyl aminomethane–hydrochloric acid (Tris–HCl) buffer solution and measuring the change of pH values and concentrations of Mg ions. The antibacterial activity of the scaffolds was investigated by the spread plate method, tissue culture plate method, scanning electron microscopy and confocal laser scanning microscopy. Additionally, the cell attachment and proliferation of the scaffolds were evaluated by the cell counting kit-8 (CCK-8) assay using MC3T3-E1 cells.

**Results:**

The Mg-incorporated scaffolds degraded and released Mg ions and caused an increase in the pH value. Both PM-L and PM-H inhibited bacterial growth and biofilm formation, and PM-H exhibited higher antibacterial activity than PM-L after incubation for 24 and 48 h. Cell tests revealed that PM-H exerted a suppressive effect on cell attachment and proliferation.

**Conclusions:**

These findings demonstrated that the PLGA/Mg scaffolds possessed favorable antibacterial activity, and a higher content of Mg (20%) exhibited higher antibacterial activity and inhibitory effects on cell attachment and proliferation than low Mg content (10%).

## Background

Bone defects are often at a high risk for infection due to the lack of soft tissue coverage, destruction of blood supply, formation of local hematomas and occurrences of tissue necrosis [1]. With regard to bone-substituting materials, surfaces of these materials are not only the cell carriers, but also support microbial colonization [2]. Most bacteria will colonize on the material surface at the site of bone defects, producing a mass of polysaccharide-protein complexes to encapsulate bacteria, thereby forming biofilms [3]. Biofilms are highly resistant to the host immune system and antibiotics [4]. Once infection occurs, the blood supply to bone is severely damaged, and osteonecrosis will occur, which makes it difficult to recruit immune cells and osteoblasts to the infected site [5]. In addition, it is difficult for antibiotics to reach effective concentrations at the infected site, which eventually leads to either delayed healing or nonhealing of the bone defect.

Various artificial bone-substituting materials have been widely used in the clinic due to the limited source of autologous bone grafts and the incompletely eliminated immunogenicity of allogeneic bones. Poly(lactide-*co*-glycolic acid) (PLGA) has been widely applied as sutures, vascular stents and bone scaffolds since it is biocompatible and its degradation rate and mechanical properties can be easily controlled by varying the copolymer ratio of lactic to glycolic acid, and it has the advantage of being capable of delivering drugs, proteins and growth factors to enhance bone healing in orthopedic applications [6, 7]. However, the acidic by-products produced during the degradation of PLGA can lead to increased inflammation and can hamper protein and growth factor delivery; in addition, the mechanical strength of PLGA is insufficient [8]. Presently, most bone-substituting materials focus only on bone repair in a sterile environment without considering the importance of preventing infection around implants.

One of the essential trace elements in the human body and the most abundant intracellular divalent cation is magnesium (Mg), and Mg plays an important role in protein and nucleic acid synthesis as well as many other cellular functions [9]. Magnesium and its alloy have been evaluated for orthopedic [10], cardiovascular and ureteral stent applications [11], owing to their good biocompatibility, unique biodegradability and satisfactory mechanical properties [12]. Studies [13, 14] recently reported that Mg also possesses antibacterial function due to the increase in pH value during its degradation. Brown et al. [15] prepared porous PLGA/Mg scaffolds using a solvent casting and salt leaching method. They found that incorporation of varying amounts of Mg (9, 20 and 50 wt%) into PLGA scaffolds increased the compressive strength and modulus. Additionally, extracts of medium from the degraded PLGA/Mg scaffolds increased the proliferation of bone marrow stromal cells in vitro and enhanced osteogenesis in vivo.

**Fig. 1:**
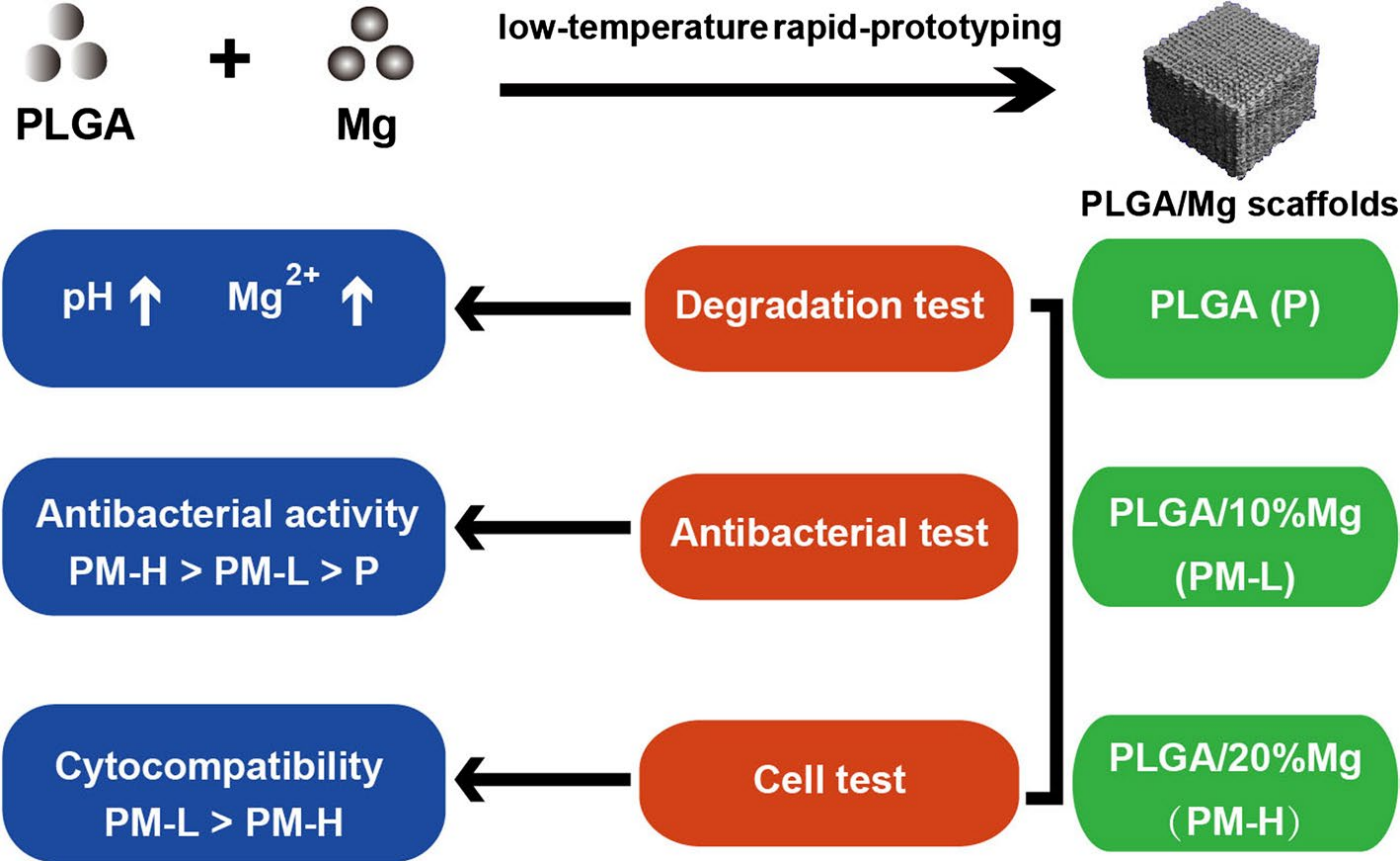
Schematic diagram of the preparation and evaluation of the PLGA/Mg scaffolds

To the best of our knowledge, no relevant studies have reported the antibacterial activity of PLGA/Mg scaffolds prepared by the low-temperature rapid-prototyping (RP) technique. We designed this study to preliminarily establish a scaffold system with favorable antibacterial activity and biocompatibility that may be used as a bone-substituting material to repair bone defects and to prevent infection. Different Mg contents were incorporated into the PLGA matrix to fabricate porous PLGA/Mg composite scaffolds using the low-temperature RP technique. The antibacterial activity and cytocompatibility of these Mg-incorporated scaffolds were investigated (Fig. 1).

## Results

**Fig. 2:**
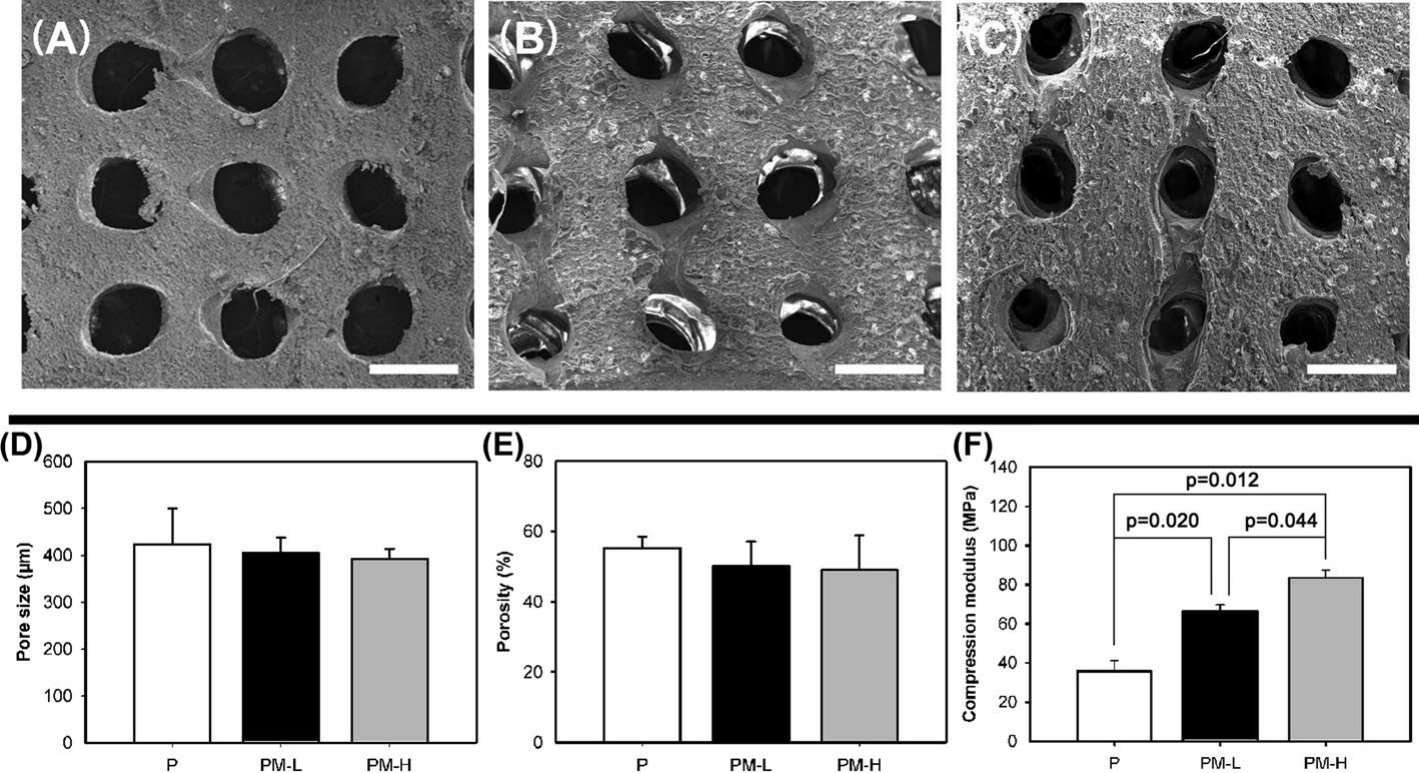
SEM images, pore size, porosity and compression modulus of the scaffolds: **a** PLGA scaffold; **b** PLGA/10% Mg scaffold; **c** PLGA/20% Mg scaffold; **d** pore size, **e** porosity, and **f** compression modulus. The scale bars represent 500 μm

The scanning electron microscopy (SEM) images of the prepared scaffolds are shown in Fig. 2. All scaffolds displayed a porous structure on the surface. For topographical difference, PLGA/10% Mg (Fig. 2b) and PLGA/20% Mg (Fig. 2c) seemed rougher than PLGA (Fig. 2a). The characterization parameters of the scaffolds are shown in Fig. 2. The pore sizes and porosity of P, PM-L and PM-H showed no significant difference (*p* > 0.05, Fig. 2d, e). With increasing Mg content, the compression moduli of the scaffolds increased. The compression modulus of PM-H (83.6 ± 3.7 MPa) and PM-L (66.5 ± 3.2 MPa) was obviously higher than that of PLGA (35.7 ± 5.5 MPa), and the compression modulus of PM-H was obviously higher than that of PM-L (*p* < 0.05).

**Fig. 3:**
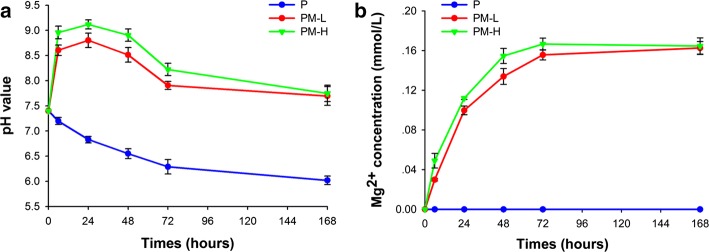
The degradation of the scaffolds after immersion into Tris–HCl buffer solution for 168 h: **a** change in pH value, and **b** change in Mg^2+^ concentration

The Mg-incorporated scaffolds degraded to cause changes in the pH value and Mg^2+^ concentration, as shown in Fig. 3. The pH value decreased gradually with the degradation of the PLGA scaffold within 168 h. In contrast, the pH value of the PM-L and PM-H scaffolds increased within 24 h and decreased slowly from 24 to 168 h. At 24 h, the pH values of the Mg-based scaffolds were highest, which was 8.80 ± 0.14 (PM-L) and 9.12 ± 0.09 (PM-H), respectively. Except for the pH value, the concentration of Mg ions increased along with the degradation of the Mg-based scaffolds (Fig. 3b). After 72 h, the Mg^2+^ concentrations of PM-L and PM-H reached a plateau stage, indicating a relatively slow degradation. The pH values and Mg^2+^ concentrations of PM-H were all higher than those of PM-L at 6, 24, 48 and 72 h (*p* < 0.05).

**Fig. 4:**
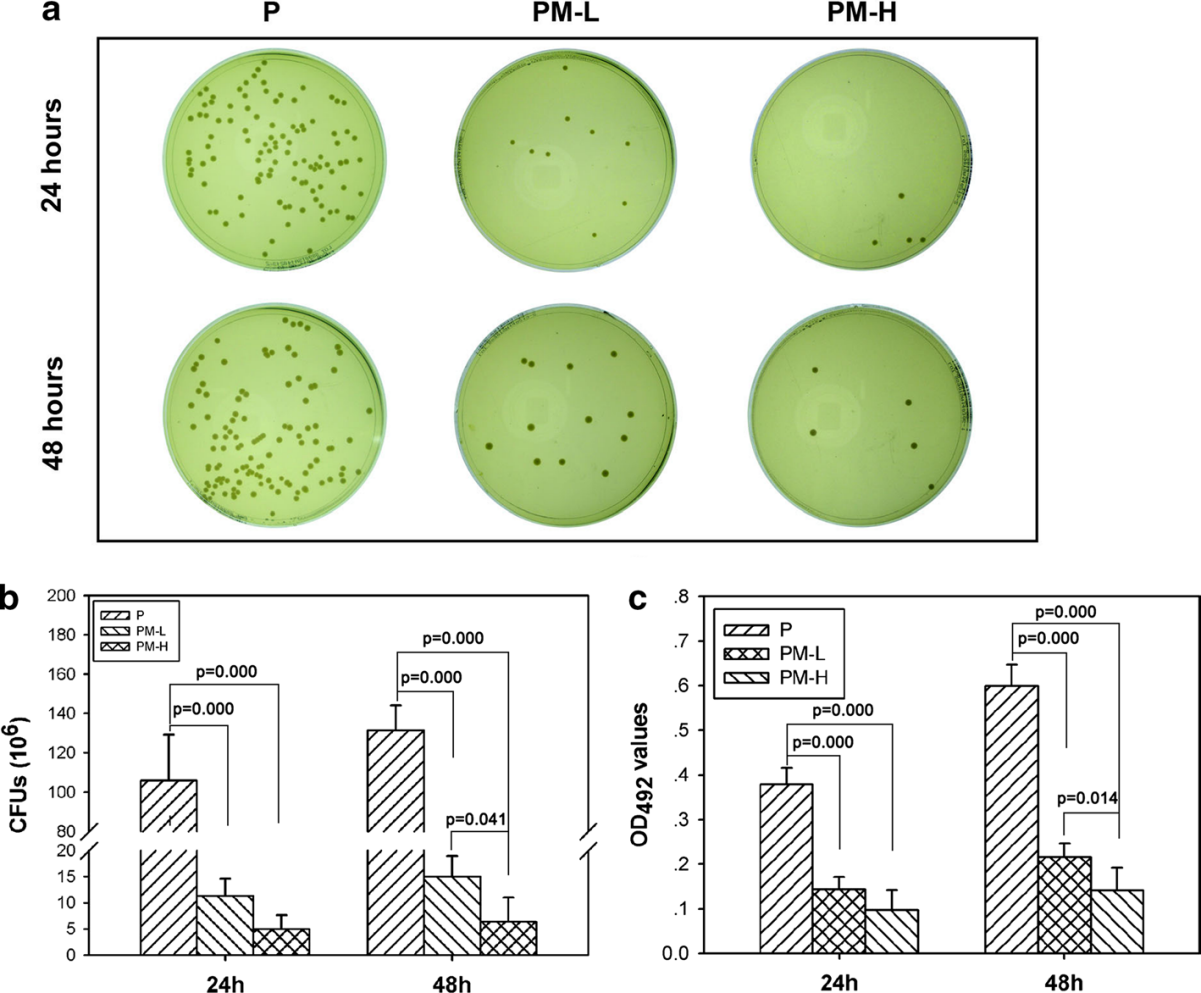
The number of viable bacteria on the surface of different specimens at 24 and 48 h as determined by the spread plate method and biofilm formation assay as determined by the TCP method: **a** representative images of TSA with bacterial colonies on the surfaces of different specimens; **b** quantitative analysis of viable bacteria; **c** quantitative analysis of biofilm formation

The bacterial growth on the scaffold surface after incubation for 24 and 48 h was evaluated by the spread plate method, which is shown in Fig. 4. The colonies on groups PM-L and PM-H seemed much less than those on group P at both time points (Fig. 4a). Quantitative analysis of the colony forming units (CFUs) on different material surfaces showed that the CFUs on groups PM-L and PM-H were apparently less than those on group P at 24 and 48 h (*p* < 0.05) (Fig. 4b). The CFUs on group PM-H were significantly less than those on group PM-L at 48 h (*p* < 0.05), but the CFUs between these two groups did not differ significantly at 24 h (*p* > 0.05). The results of the biofilm formation as assessed by the tissue culture plate (TCP) method are shown in Fig. 4c. At 24 h, the optical density (OD) values of the PM-L and PM-H groups were lower than that of the P group (*p* < 0.05). At 48 h, the PM-L group exhibited clearly lower OD values than the P group, and PM-H exhibited clearly lower OD values than the PM-L and P groups (*p* < 0.05), which was consistent with the results obtained by the spread plate method.

**Fig. 5:**
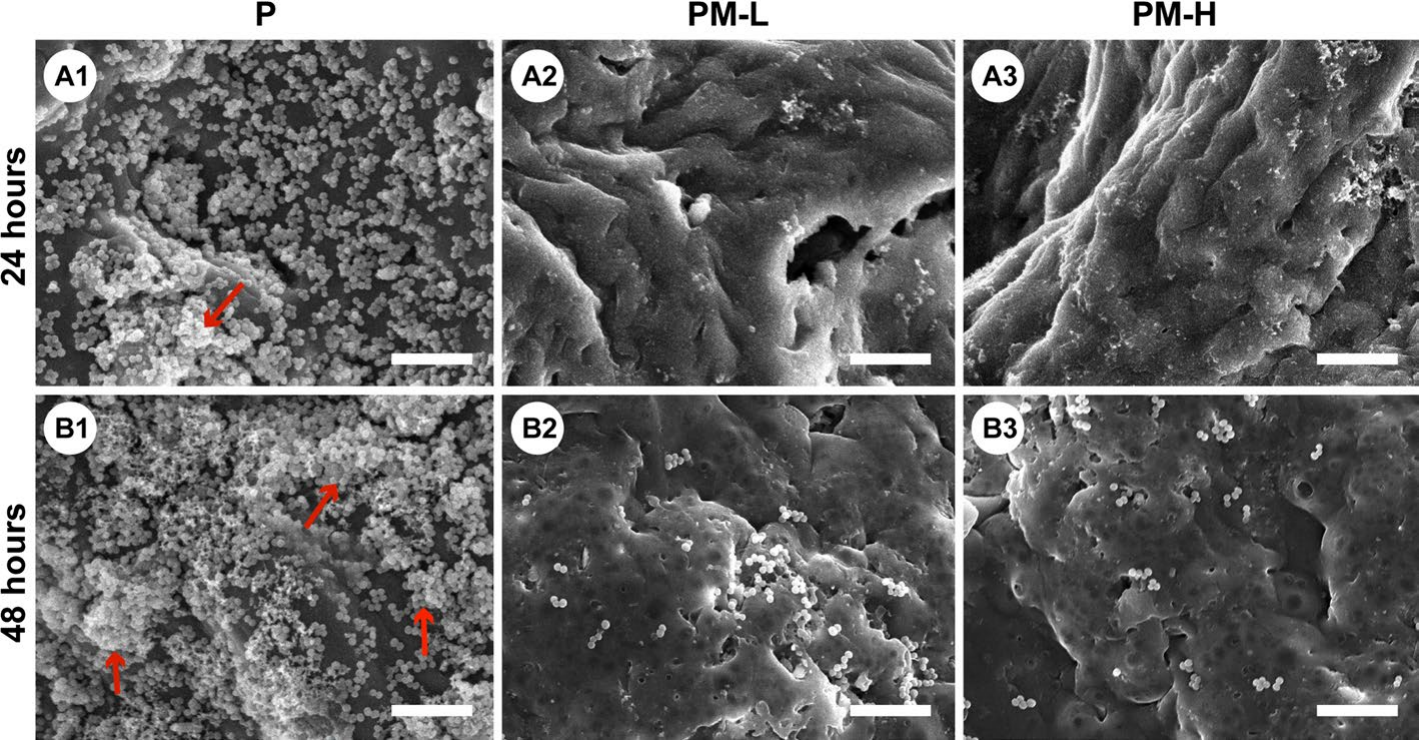
Representative SEM images showing bacterial growth and biofilm formation on the surfaces of specimens. The red arrows indicate the formed biofilm. The scale bar is 10 μm

The bacteria on different material surfaces incubated for 24 and 48 h were observed by SEM and are presented in Fig. 5. At 24 h, the bacteria on the surface of group P were exuberantly growing in clustered and agglomerate conditions, and a fraction of biofilms had formed (red arrow in Fig. 5a1), while bacteria were rarely observed on the surfaces of the PM-L and PM-H groups. After incubation for 48 h, a mass of biofilms had formed and covered most areas of the surface of group P (red arrows in Fig. 5b1); however, only some scattered spherical bacteria were observed on the surface of groups PM-L and PM-H.

**Fig. 6:**
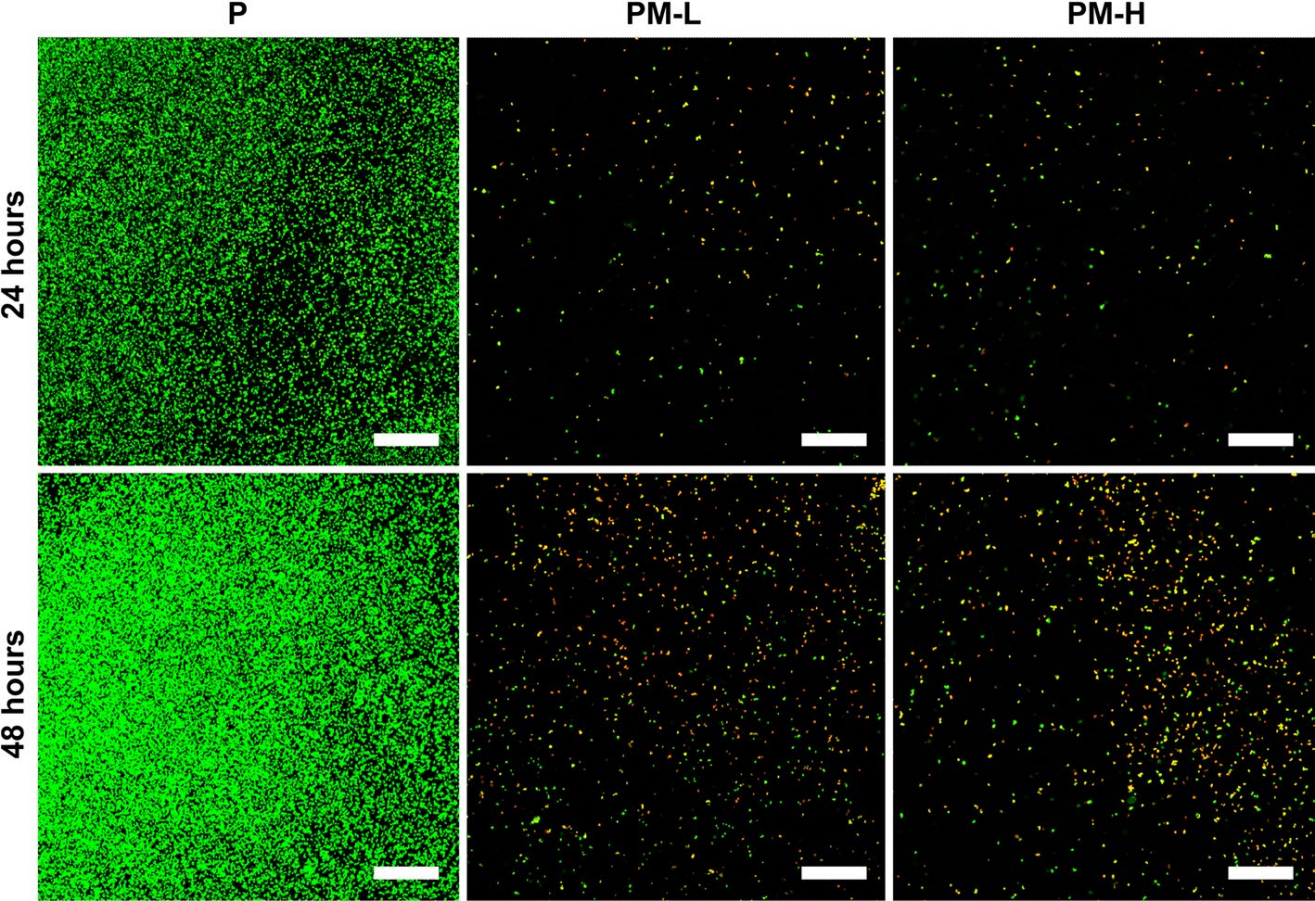
CLSM images showing live and dead bacteria in different groups. Live cells appeared green, and dead cells appeared red under CLSM. The scale bars are equal to 50 μm

Live and dead bacteria were observed by confocal laser scanning microscopy (CLSM) and are shown in Fig. 6. It could be observed that an evident biofilm had formed at 24 h on group P, and the biofilm continued to be more mature after incubation for 48 h. In contrast, in the PM-L and PM-H groups, few sporadic green fluorescent markers were observed at 24 h, indicating that live bacteria were limited, and no biofilm was formed. Moreover, many apparent red fluorescence markers and a few green fluorescence markers could be found on groups PM-L and PM-H at 48 h, indicating that a large number of bacteria were killed so that no biofilm could be formed.

**Fig. 7:**
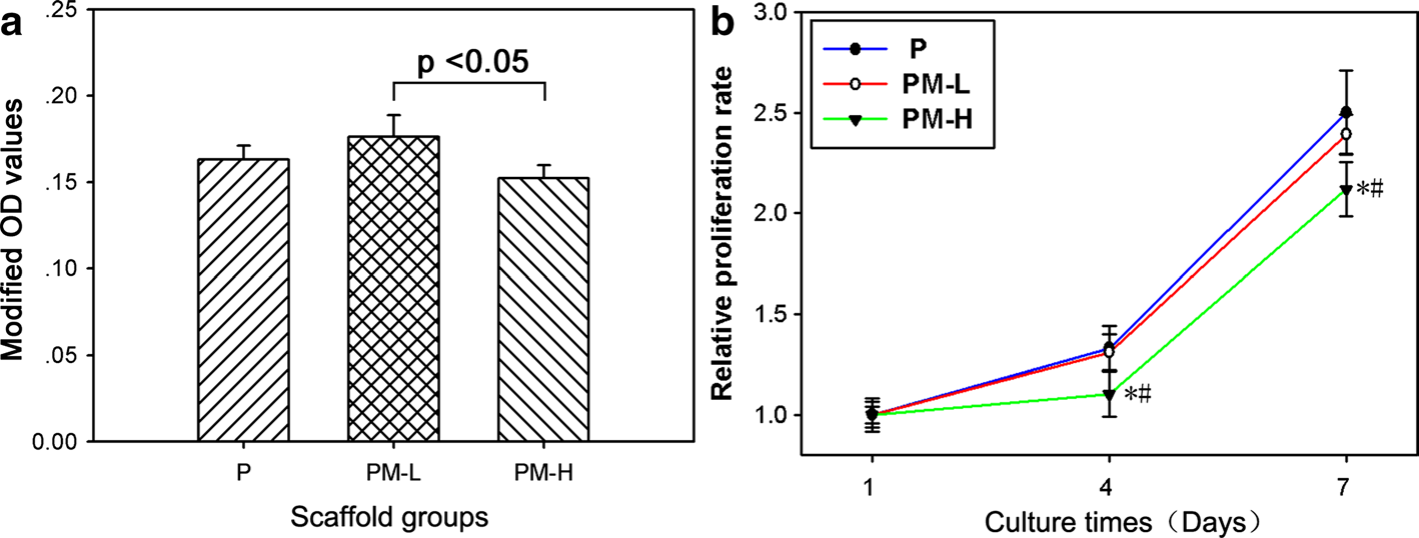
Cell attachment of MC3T3-E1 cells on the scaffolds at 6 h (**a**) and cell proliferation of MC3T3-E1 cells on the scaffolds (**b**). Modified OD values are ODs measured at 450 nm subtracted by ODs measured at 620 nm. The modified OD values at days 4 and 7 were normalized to those at day 1. * denotes *p* < 0.05 compared with the P group, and # denotes *p* < 0.05 compared with the PM-L group

Cell attachment was assessed by the cell counting kit-8 (CCK-8) assay at 6 h and is shown in Fig. 7a. The number of attached cells on the PM-H group was significantly less than that on the PM-L group (*p* < 0.05). The differences of the attached cells between the PM-H and P groups and between the PM-L and P groups were not statistically significant (*p* > 0.05). Figure 7b presents MC3T3-E1 cell proliferation on different specimens at days 1, 4 and 7. The modified OD values at 4 and 7 days were normalized to those at day 1. The relative proliferation rate of MC3T3-E1 cells on group PM-H was found to be significantly lower than those on groups P and PM-L (*p* < 0.05) at both days 4 and 7. Furthermore, there was no significant difference in the relative proliferation rate between the PM-L and P groups at days 4 and 7 (*p* < 0.05).

## Discussion

Bone defects are a troublesome problem in the orthopedic clinics and are often at risk of infection. An implanted bone substitute may become a carrier for bacterial adhesion and colonization [16]. It is very important to design new bone substitutes to repair bone defects and prevent infection. Mg has been increasingly researched in the past decade and has been found to possess antibacterial functions due to the increase in the pH value during its degradation [17].

In the present study, we designed a Mg-incorporated scaffold with PLGA as the matrix using a low-temperature rapid-prototyping technique to repair small critical-sized bone defects with antibacterial activity. It has been reported that the addition of Mg particles to the PLGA scaffolds would increase the compressive strength and modulus compared with PLGA-only scaffolds [18, 19]. In our study, the compression moduli of the PM-H and PM-L scaffolds increased by 134.2% and 86.3%, respectively, compared to the PLGA-only scaffolds (Fig. 2f). The PLGA matrix will benefit from the higher strength and modulus of the Mg particles, and the prepared PLGA/Mg scaffold possessed certain anti-compression ability to resist compressive stress in vivo.

The majority of bacteria that enter the bone defect site will colonize on the implant surface or necrotic tissues and will produce many polysaccharide-protein complexes to wrap bacteria and form biofilms. The ability of bacteria to develop antibiotic resistance and colonize on the abiotic surfaces by forming biofilms is a major cause of orthopedic infections [4]. The ability to inhibit biofilm formation is crucial when evaluating the antibacterial activity of materials. The results of the antibacterial tests showed that the PLGA/Mg scaffolds could inhibit bacterial growth and biofilm formation at 24 and 48 h, and the inhibitory effect was more obvious in the group with higher Mg content (Fig. 4). From SEM and CLSM images, it could be seen that scattered bacteria were found on the surface of the Mg-incorporated groups, and concentrated bacteria forming apparent biofilm were found on the surface of Mg-absent group. Most organisms have a pH range in which preferential growth occurs [20]. For example, bacteria can generally live in an environment with a pH range of 6.0–8.0, in which bacteria can maintain a cytoplasmic pH that is compatible with the optimal functional and structural integrity of cytoplasmic proteins [21]. In our present study, the pH values of the PLGA/Mg groups were higher than that of PLGA within 48 h, and the PLGA/20% Mg scaffold with higher Mg content caused a higher pH value than the PLGA/10% Mg scaffold (Fig. 3a). In other words, higher Mg content caused higher pH and possessed stronger antibacterial activity. In theory, higher Mg content means stronger antibacterial activity, but excessive alkaline environment is detrimental to cells. Seeking an appropriate Mg content that is beneficial for osteoblasts and bacteriolytic for bacteria is vitally important.

Many studies have reported the mechanism of Mg-based materials for inhibiting bacterial growth and biofilm formation. The corrosion mechanisms that occur when Mg is exposed to an aqueous environment are as follows [22]:

$${\text{Mg }} + {\text{ 2H}}_{ 2} {\text{O}} \to {\text{Mg}}^{ 2+ } + {\text{ 2OH}}^{ - } + {\text{ H}}_{ 2}$$

The Mg-incorporated scaffolds degraded to cause increased pH values and Mg^2+^ concentrations, which are consistent with our results (Fig. 3). Lock et al. [23] reported that the degradation of Mg in artificial urine led to an increase in ionic Mg concentrations and an increase in the pH of the solutions. Both of these characteristics potentially contributed to the antibacterial properties of Mg-based materials. However, Robinson et al. [13] considered that the degradable characteristic of Mg in a physiological solution could result in rapid increases in both the Mg^2+^ concentration and pH, and the latter should be responsible for the antibacterial function of Mg. In our previous study, we found that Mg-based scaffolds degraded to cause an alkaline pH, increased Mg^2+^ concentration, and increased osmolality. The increased pH value was proven to be the primary cause for antibacterial activity of Mg [24]. A study from Germany proved that the proliferation of *S. aureus* and *P. aeruginosa* was suppressed in the presence of metallic Mg and in aqueous Mg corrosion extracts. The alkaline pH was sufficient in providing antibacterial effects, which were completely abolished when the pH of the corrosion supernatants was neutralized [25]. In this study, the prepared PLGA/Mg scaffold released Mg ions and increased the pH with its degradation. Based on the reported research together with the results in this study, the reason for the antibacterial activity of PLGA/Mg scaffolds should be caused by the alkaline environment produced by the Mg degradation of the PLGA/Mg scaffolds.

Biocompatibility is very important when evaluating a biomaterial. The cell attachment and proliferation of MC3T3-E1 cells on the scaffolds were evaluated. It was found that cell attachment to PM-H was lower than that of PLGA after 6 h, and cell proliferation on PM-H scaffolds was lower than the PLGA and PM-L groups at days 4 and 7, which meant higher Mg content (20%) may be harmful to cell growth. Mg-based scaffolds exhibited increased pH values after Mg was degraded. However, an environment that is too alkaline would inhibit cell growth and can even kill cells [26]. The alkaline pH caused by the degradation of PM-H scaffolds was favorable to inhibiting bacterial growth and deleterious to cell growth. That is, the PM-H scaffold was not beneficial for bone repair.

Based on the results of this study, higher Mg content (20%) was proven to possess stronger antibacterial activity than lower Mg content (10%). However, higher Mg content was also toxic to osteoblasts. The exact incorporated Mg content, which exerts maximum antibacterial effect and minimum cytotoxic effects, is still unclear and requires further study. Furthermore, the bone-repairing ability and in vivo degradability of PLAG/Mg scaffolds need to be verified and the ability of PLGA/Mg scaffolds to achieve antibacterial effects in vivo requires further study regarding the effect of various enzymes and body fluids.

## Conclusion

A Mg-incorporated scaffold was fabricated with different contents of Mg (10 wt% and 20 wt%) and PLGA (as the matrix) by using a low-temperature rapid-prototyping technique. Although the high Mg content (20%) was found to have stronger antibacterial activity than that of the low Mg content (10%), the PLGA/20% Mg scaffold exhibited inhibitory effects on MC3T3-E1 cell attachment and proliferation. In consideration of both good antibacterial activity and favorable cytocompatibility, the PLGA/10% Mg scaffold was believed to be more suitable than the scaffold containing 20% Mg to be used as a bone substitute at critical-sized bone defect sites.

## Methods

### Preparation of scaffolds

Mg powder, with a particle size of 50 μm, was obtained from Shenzhen Tianyuan Magnesium Ltd. (China), and PLGA with an average molecular weight of 15.5 × 10^4^ Da was purchased from Shandong Institute of Medical Instruments (China). Porous PLGA/Mg scaffolds were fabricated by a low-temperature RP technique. Two different scaffolds with different contents of Mg [PLGA/10 wt% Mg (PM-L) and PLGA/20 wt.% Mg (PM-H)] were prepared. PLGA scaffolds (P) served as the control group. The fabrication process was the same as detailed in our previous study [24]. The surface topographies of the scaffolds were observed by scanning electron microscopy (SEM; S-4800, Hitachi, Tokyo, Japan). The samples were scanned with microcomputed tomography (Micro-CT; μCT80, SCANCO, Bern, Switzerland) with a resolution of 21 μm, and the pore size and porosity were calculated. The compression modulus of the scaffolds (*n* = 5) was measured by a mechanical test machine (Instron 5567, Norwood, Massachusetts, USA).

### Degradation test

The scaffolds were soaked in trihydroxymethyl aminomethane–hydrochloric acid (Tris–HCl) buffer solution (1 M, pH = 7.40) with a surface area-to-volume ratio of 0.1 cm^−1^ at 37 °C for 6, 24, 48, 72 and 168 h. At each time point, the samples were removed, and the concentrations of Mg ions in the soaked solutions were measured by inductively coupled plasma atomic emission spectroscopy (ICP-AES; Varian, Palo Alto, California, USA), and the pH values of the soaked solutions were measured by a flat membrane microelectrode (PB-10, Sartorius, Germany).

### Quantitative analysis of antibacterial activity

*Staphylococcus epidermidis* (ATCC35984) was selected as the testing strain. The spread plate method was used to investigate bacterial growth. The inoculum of the strain was prepared by adjusting the concentration to 10^6^ CFUs/mL in trypticase soy broth (TSB). A volume of 500 μL of the suspension with 10^6^ CFUs/mL bacteria was added to wells that contained scaffold specimens and incubated at 37 °C in a humidified atmosphere for 24 or 48 h. Then, the specimens were gently washed with sterile phosphate-buffered saline (PBS) three times to remove the loosely adherent bacteria, and the adherent bacteria on the scaffolds were removed by ultrasonication in a 150 W and 50 Hz ultrasonic bath (B3500S-MT, Branson Ultrasonics Co., Shanghai, China) for 20 min. The solutions, collected after ultrasonication, experienced a tenfold dilution process. The 10^6^-, 10^7^-, and 10^8^-fold dilute solutions were plated in triplicate onto tryptone soy agar (TSA) and then incubated at 37 °C in a humidified atmosphere for 24 h. The number of colonies on the TSA was counted. The ultimate colony forming units (CFUs) were the number of colonies on the TSA multiplied by the dilution ratio.

The tissue culture plate (TCP) method was used to quantitatively detect biofilm formation. After coincubation for 24 and 48 h, the specimens were gently washed with PBS three times and then fixed with 2.5% glutaraldehyde for 30 min at 4 °C and dried at 60 °C for 30 min. Afterwards, the specimens were stained with 500 μL of 0.1% crystal violet (CV; Sigma-Aldrich, St. Louis, MO, USA) solution at room temperature for 15 min. The samples were rinsed thrice with PBS and dried at 37 °C for 2 h. The stained CV was dissolved in 500 μL of 2% glacial acetic acid (Sigma-Aldrich) for 15 min with agitation at 200 rpm. The biofilms were quantified by measuring the optical density (OD) using a microplate reader (Synergy HT, Biotek, Winooski, VT, USA) at a wavelength of 492 nm.

### SEM observation

A volume of 500 μL of the suspension with 10^6^ CFUs/mL bacteria was added to wells that contained scaffold specimens and incubated at 37 °C in a humidified atmosphere for 24 and 48 h. Then the specimens were gently washed three times with PBS, fixed in 2.5% glutaraldehyde for 30 min, washed three times with PBS again, and dehydrated with a series of graded ethanol solutions. Then, the specimens were air dried, sputter coated with gold, and observed using SEM (S-4800, Hitachi, Tokyo, Japan).

### Confocal laser scanning microscopy (CLSM) observation

Using bacterial live/dead staining, bacteria were stained with green fluorescent SYTO 9 and red fluorescent propidium iodide. A volume of 500 μL of the suspension with 10^6^ CFUs/mL bacteria was added to wells that contained scaffold specimens and incubated at 37 °C in a humidified atmosphere. After coincubation for 24 and 48 h with bacteria, the specimens were stained with 300 μL of combination dye (Live/Dead BacLight bacteria viability kits; Molecular Probes Life Technologies, Carlsbad, CA, USA) and observed with CLSM (Leica TCS SP2, Heidelberg, Germany). Live bacteria appear as fluorescent green, while the dead bacteria appear as fluorescent red.

### Cytocompatibility

MC3T3-E1 cells were used to investigate in vitro cytocompatibility in this study. The cells were cultured in Dulbecco’s modified Eagle’s medium (DMEM; HyClone, Thermo Fisher Scientific Inc., Miami, Florida, USA), supplemented with 10% fetal bovine serum (FBS; GibcoBRL, Grand Island, New York, USA) and 1% penicillin and streptomycin sulfate(100 U/mL, GibcoBRL) at 37 °C in a humidified atmosphere with 5% CO_2_. The culture medium was changed every 3 days. A cell counting kit-8 (CCK-8) assay was used to analyze cell attachment on the scaffolds after 6 h. The MC3T3-E1 cells were seeded in 48-well plates containing the scaffold specimens at a density of 6 × 10^4^/cm^2^ (4.8 × 10^4^/well), with wells containing the scaffold specimens and DMEM set up as a negative control. After coincubation for 6 h, the specimens were transferred to a fresh 48-well plate and gently rinsed three times with PBS to remove the unattached cells. A volume of 30 μL of CCK-8 solution (Dojindo Molecular Technologies Inc., Kumamoto, Japan) was added to each well and incubated for 3 h at 37 °C. Then, the OD value was read at 450 nm and 620 nm using a microplate reader (Synergy HT, Biotek, Winooski, Vermont, USA). The mean OD that was obtained from the negative control was subtracted from the ODs of the test groups. Cell proliferation was also investigated using the CCK-8 assay after 1, 4, and 7 days. The seeding density of cells was 2 × 10^4^/cm^2^ (1.6 × 10^4^/well). The OD values at days 3 and 7 were normalized to the values at day 1.

### Statistical analysis

All data were expressed as the mean ± standard deviation (SD). All experiments were conducted in triplicate and repeated three times. All statistical analyses were performed using SPSS software (version 13.0). The results were tested using one-way analysis of variance (ANOVA) and least significant difference (LSD) tests to determine the level of significance, with *p *< 0.05 significant.

## Data Availability

Data sharing not applicable to this article as no datasets were generated or analyzed during the current study.

## Abbreviations

Mg: Magnesium
PLGA: Poly(lactide-*co*-glycolic acid)
P: PLGA
PM-L: PLGA/10 wt% Mg with low Mg content
PM-H: PLGA/20 wt% Mg with high Mg content
Tris–HCl: Trihydroxymethyl aminomethane–hydrochloric acid
CCK-8: Cell counting kit-8
RP: Rapid-prototyping
SEM: Scanning electron microscopy
CFUs: Colony forming units
TCP: Tissue culture plate
OD: Optical density
CLSM: Confocal laser scanning microscopy
Micro-CT: Microcomputed tomography
ICP-AES: Inductively coupled plasma atomic emission spectroscopy
TSB: Trypticase soy broth
PBS: Phosphate-buffered saline
TSA: Tryptone soy agar
CV: Crystal violet
DMEM: Dulbecco’s modified Eagle’s medium
FBS: Fetal bovine serum
SD: Standard deviation
ANOVA: Analysis of variance
LSD: Least significant difference
